# Supplementary material for: Disease-induced resource constraints can trigger explosive epidemics
Source: Sci Rep. 2015 Nov 16;5:16571. doi: 10.1038/srep16571 (PMC4644972; doi:10.1038/srep16571)
Supplement: Supplementary Materials [file srep16571-s1.pdf]

Supplementary Material: Disease-induced resource constraints can trigger explosive epidemics

L. Böttcher,[1] O. Woolley-Meza,[2,*] N. A. M. Araújo,[3] H. J. Herrmann,[1,4] and D. Helbing[5]

[1]*Computational Physics for Engineering Materials, IfB, ETH Zurich, Wolfgang-Pauli-Strasse 27, CH-8093 Zurich, Switzerland*

[2]*Computational Social Science, ETH Zurich, Clausiusstrasse 37, CH-8092 Zurich, Switzerland*

[3]*Departamento de Física, Faculdade de Ciências, Universidade de Lisboa, P-1749-016 Lisboa, Portugal, and Centro de Física Teórica e Computacional, Universidade de Lisboa, Avenida Professor Gama Pinto 2, P-1649-003 Lisboa, Portugal*

[4]*Departamento de Física, Universidade Federal do Ceará, 60451-970 Fortaleza, Ceará, Brazil*

[5]*Computational Social Science, ETH Zurich, Clausiusstrasse 50, CH-8092 Zurich, Switzerland*

[*] owoolley@ethz.ch

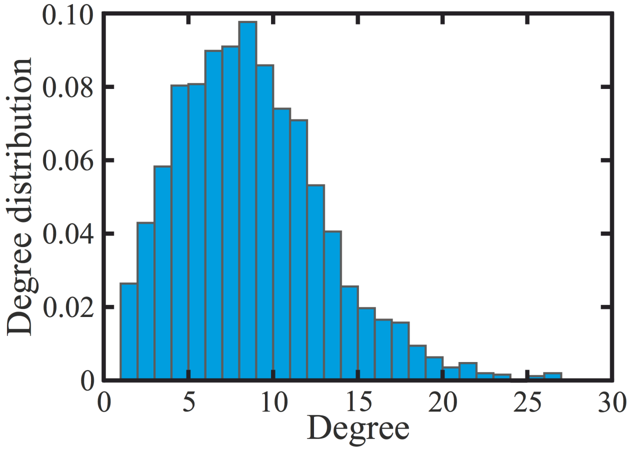

Figure S1. **Degree distribution of the school friendship network.** The network has 2539 nodes, 20910 edges, and average degree $\langle k \rangle \approx 8.2355$.

I. FRIENDSHIP NETWORK

We implemented the budget-Susceptible-Infected-Susceptible model on a real network. A school friendship network was considered with 2539 nodes and 20910 edges [1], obtained from a public data set from Add Health [2]. Figure S1 shows the degree distribution of the network, with an average degree $\langle k \rangle \approx 8.2355$.

II. SQUARE LATTICE

The dependence of the critical time (t^*) on the distance to the critical cost $(c - c^*)$ is shown in Fig. S2. A power law is also observed with an exponent -0.98 ± 0.03.

III. BUDGET FUNCTION

For simplicity, in the main text, we only consider the case where the budget function $f(b)$ is the Heaviside step function, $\Theta(b)$. This function is discontinuous for $b = 0$. To show that the abrupt transition to the high epidemic regime is not a consequence of this discontinuity, here we also consider a continuous budget function defined as

$$f(b) = \begin{cases} 0 & , b < 0 \\ 1 - \exp(-b) & , b \geq 0 \, . \end{cases} \tag{1}$$

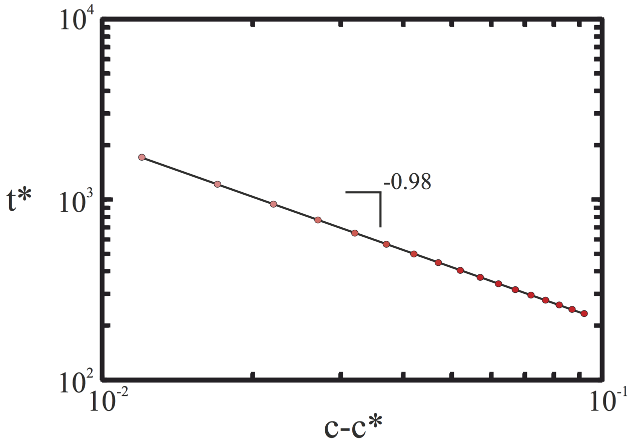

Figure S2. **Scaling of the critical time with the distance to the critical cost on a square lattice.** A power law is observed, $t^* \sim (c - c^*)^b$, with $b = -0.98 \pm 0.03$. Simulations are performed on a lattice with 1024^2 sites and $q = q_b = 0.8$. Results are averages over 3600 samples.

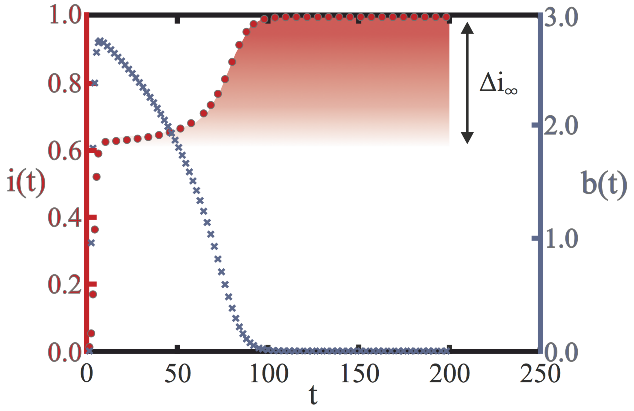

Figure S3. **Change in regime at the critical time.** Evolution of the fraction of infected individuals $i(t)$ (red circles) and budget $b(t)$ (blue crosses), for a fixed healing cost ($c = 0.833$) above the critical cost, in the mean-field limit with the budget function defined by Eq. (1). The data points are for numerical data obtained with the Runge-Kutta method of fourth order. The recovery rate is $q = 0.8$, the average degree $k = 4$, and the infection rate is $p = 0.5$.

Using this budget function we numerically integrate the mean-field equations (Eqs. (1) and (2) in the main text with $q_0 = 0$ and therefore $q = q_b$), using the Runge-Kutta method of fourth order. As shown in Fig. S3, for certain values of the model parameters, a transition to a high epidemic regime is observed. and this transition is discontinuous in the parameter space, as evident from Fig. S4.

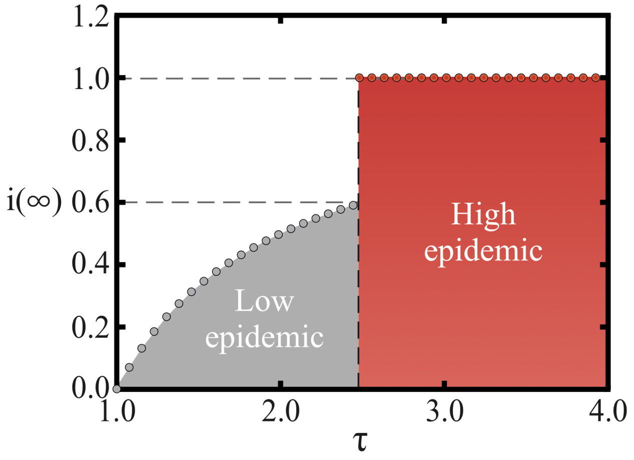

Figure S4. **Discontinuous transition from low to high epidemic regime.** Asymptotic fraction of infected individuals $i(\infty)$ in the mean-field limit, for the budget function defined by Eq. (1), for fixed cost ($c = 0.833$), average degree ($k = 4$), and recovery rate ($q = q_b = 0.8$) as a function of the infection rate p. A discontinuous transition from low to high epidemic regime is observed.

IV. MODEL WITH PARTIAL COSTLESS HEALING

We can analyze the final state of a more general model with SIS dynamics and an additional budget healing contribution, described by Eqs. (1) and (2) in the main text, with $q_0 > 0$. As in the main text we define $\tau_{q_0} = kp/q_0$ and notice that for $\tau_{q_0} \leq 1$ the fraction of the infected tends to zero, independent of the budget contribution. Therefore, we can focus on the $\tau_{q_0} > 1$ regime and consider the different cases: (i) the initial fraction $i(0)$ is smaller or equal than $i_0(\infty) = 1 - (q_0 + q_b)/(kp) = 1 - \tau^{-1}$ for $\tau > 1$ and (ii) the initial fraction is greater than $i_0(\infty)$ independent of τ. Demanding a stationary budget function $db(t)/dt = 0$ implies for the first case ($i(0) \leq i_0(\infty)$, $\tau > 1$):

$$c^* = \frac{q_0 + q_b}{q_b[kp - (q_0 + q_b)]}. \tag{2}$$

In the second case the critical cost needs to be large enough to keep the fraction of the infected at the initial level. (As we shall see later, this is not always possible.) To do this we search for the stationary state of Eq. (5) of the main text at the initial level $i(0)$ (where $i(0) > i_0(\infty)$, independent of τ):

$$c^* = \frac{1 - i(0)}{i(0)[kp(1 - i(0)) - q_0]}. \tag{3}$$

The first case is a special case of the second one, as can be seen by setting $i_{max} = \max_t(i_0(t))$ and defining c^* in general as:

$$c^* = \frac{1 - i_{max}}{i_{max}[kp(1 - i_{max}) - q_0]}. \quad (4)$$

We can recover Eq. (2) by assuming $i_{max} = i_0(\infty) = 1 - \tau^{-1}$. Eq. (4) only holds when $i(0) < 1 - \tau_{q_0}^{-1}$ or c^* is negative. Otherwise we cannot keep the fraction of the infected at the initial level and another equilibrium will be reached. We note that this equilibrium must be higher since it cannot be the case that the system with the higher initial infection level has a smaller critical cost c^*.

When $c > c^*$ for all values of τ the system changes according to Eq. (5) in the main text, which has the stationary points given by Eq. (8) of the main text. It is possible to further analyze these stationary states using the boundary condition $i(0)$ and the fact that $c > c^*$. We can for instance give some general arguments about the relative sizes and the stability of solutions under the constraint $c > c^*$. For the $\tau > 1$ regime, one can show that the inequality $i(\infty)^- < i_0(\infty) < i(\infty)^+$ is always fulfilled (at $c = c^*$ by definition $i_0(\infty) = i(\infty)^+$ in Eq. (2) and $i(0) = i(\infty)^-$ in Eq. (3)). The phase spaces of Eq. (5) of the main text (illustrated in Fig. 6 of the main text) and the underlying SIS model illustrate the stability of $i(\infty)^+$. However, with certain parameters it is also possible that the fraction of the infected stays at the initial level (Eq. (4)) independent of τ. However, we note that in these cases c^* must be smaller than that given in Eq. (2) and thus the equilibrium infection level can not fall below $i_0(\infty)$. We can conclude that when $c > c^*$ and $\tau_{q_0} > 1$, there is always an explosive epidemic transition from a lower to a higher epidemic level.

---

[1] M. C. González, H. J. Herrmann, J. Kertész, and T. Vicsek, Physica A **379**, 307 (2007).

[2] A program project designed by J. Richard Udry, Peter S. Bearman, and Kathleen Mullan Harris, and funded by a grant from the National Institute of Child Health and Human Development (P01-HD31921). For data les from Add Health contact Add Health, Carolina Population Center, 123 W. Franklin Street, Chapel Hill, NC 27516-2524, http://www.cpc.unc.edu/addhealth .
